# The impact of adjuvant therapies on patient survival and the recurrence patterns for resected stage IIa–IVa lower thoracic oesophageal squamous cell carcinoma

**DOI:** 10.1186/s12957-018-1516-1

**Published:** 2018-11-07

**Authors:** Yichun Wang, Liyang Zhu, Wanli Xia, Liming Wu, Fan Wang

**Affiliations:** 1 Department of Radiation Oncology, The First Affiliated Hospital of Anhui Medical University, No.218, Jixi Road, Hefei, 230022 Anhui People’s Republic of China; 2 Department of Thoracic Surgery, The First Affiliated Hospital of Anhui Medical University, Hefei, 230022 Anhui People’s Republic of China

**Keywords:** Oesophageal neoplasm, Radiotherapy, Recurrence, Lymph node, Clinical target volume

## Abstract

**Background:**

This study evaluated the impact of adjuvant therapies on patient survival and disease recurrence patterns to identify an effective adjuvant therapy for resected lower thoracic oesophageal squamous cell carcinoma (LTESCC).

**Methods:**

Clinical data of 127 patients with stage IIa-IVa LTESCC with a minimum 2-year follow-up after oesophagectomy were analysed. The survival and recurrence patterns were compared among patients who received adjuvant radiotherapy, adjuvant chemotherapy, adjuvant chemoradiotherapy, or surgery alone.

**Results:**

Eighty-eight patients (69.3%) were identified as having disease recurrence. The regional lymph node recurrence rate was 57.5%, and the recurrence rates were high in the lower neck, upper mediastinum, and upper abdomen. Compared to surgery alone, adjuvant radiotherapy or chemoradiotherapy significantly decreased the recurrence rate (*p* < 0.05). Adjuvant chemoradiotherapy significantly improved overall survival, disease-free survival, and locoregional recurrence-free survival compared to surgery alone (*p* = 0.01, 0.01, and 0.00, respectively). Pathologically positive lymph nodes (PPLNs) in the lower mediastinum represented a potential risk factor for cervical recurrence (HR 2.97, 95%CI 1.19–7.39). Multivariable analysis showed that postoperative radiotherapy (HR 0.30, 95%CI 0.13–0.68) and PPLNs in the upper mediastinum (HR 3.72, 95%CI 1.30–10.67) were independent risk factors for upper mediastinal recurrence, while postoperative radiotherapy (HR 0.37, 95%CI 0.16–0.85) and PPLNs in the abdomen (HR 2.57, 95%CI 1.12–5.92) were independent risk factors for abdominal recurrence.

**Conclusion:**

Adjuvant chemoradiotherapy was the most effective adjuvant therapy for resected stage IIa-IVa LTESCC. The lower neck, upper mediastinum, and upper abdomen were high-risk regions for postoperative radiotherapy. The regions of PPLNs may be important factors for individual targets.

## Background

Despite improvements in the diagnosis and treatment of oesophageal carcinoma (EC), the overall 5-year survival rates are still very low (~ 40%) [1, 2]. There is a high incidence of lower thoracic oesophageal adenocarcinoma in western countries, while lower thoracic oesophageal squamous cell carcinoma (LTESCC) has a high incidence in Southeastern Asia [3]. The characteristics of oesophageal adenocarcinoma (EAC) are quite different from those of oesophageal squamous cell carcinoma (ESCC). The gene expression of ESCC is most semblable to that of head and neck squamous cell carcinoma, while EAC is most semblable to gastric adenocarcinoma [4]. Adjuvant chemoradiotherapy (aCRT) is associated with a significant overall survival (OS) advantage for advanced resected gastric cancer and head and neck squamous cell carcinoma [5, 6], which may be an implication for adjuvant treatment of ESCC. However, neoadjuvant chemoradiotherapy (nCRT) is recommended for advanced EC, particularly EAC [7, 8]. Adjuvant therapies appear to be less effective than are the neoadjuvant approaches, and there have been relatively few studies in recent years [8]. Only a few studies [9–11] have compared nCRT and aCRT, and only one study [11] concluded that nCRT was associated with a trend towards better OS for resectable stage II/III ESCC. The non-standardised plan of postoperative radiotherapy (PORT) should also be taken into consideration for evaluating treatment efficacy, such as controversial target volume and dose. Furthermore, surgery is still the first choice for ESCC patients in many countries. Hence, adjuvant therapies have also been studied for many years, and some good outcomes have been achieved for advanced resected ESCC, especially aCRT [12–15].

Platinum-based treatment regimens are still the standard chemotherapy for EC. There is no consensus about the clinical target of PORT for ESCC due to the wide-range, bidirectional, and skipping lymph node metastasis (LNM). Recurrence patterns after radical surgery were analysed to provide more evidence for PORT [16–21]. Local recurrence in the lower neck and upper mediastinum after radical surgery was commonly found by two-field lymphadenectomy (2FL) or three-field lymphadenectomy (3FL) for thoracic ESCC, and these regions were recommended to be encompassed within the target volume [22]. Recurrence in the upper abdomen is also common due to the main descending lymph flow with LTESCC [20, 21, 23]. Hence, a wide range of irradiation fields has been used for PORT of LTESCC in many departments, from the supraclavicular area to the upper abdominal area [14]. However, such a wide field of irradiation may lead to more treatment intolerance and complications. Moreover, the anatomy of the tumour bed and the upper abdomen changed obviously after surgery, which makes it difficult to delineate these areas for treatment. As a result, the clinical target of PORT for LTESCC seems to be more controversial compared to upper and middle thoracic ESCC.

Although the recurrence patterns of EC after radical surgery have been analysed by many researchers [16–21], comprehensive studies of LTESCC alone are limited. A proposed T-shaped field could cover over 80% of the local-regional failure for LTESCC [23], and a proposed abdominal target area was concluded for EC treatment [24]. However, no research has evaluated the efficiency of adjuvant therapies alone for LTESCC. We need more evidence for an effective clinical target of PORT, especially for areas with low recurrence rates, such as the lower mediastina, primary perigastric area, and tumour bed. Furthermore, there may be some characteristics and risk factors for predicting regional lymph node recurrence that should be taken into consideration for delineating the target treatment area. Therefore, we performed a retrospective analysis of LTESCC patients after radical surgery in our hospital to identify a more effective adjuvant therapy.

## Patients and methods

### Patients

Clinical data of patients after radical oesophagectomy for LTESCC from January 2013 to January 2016 at the First Affiliated Hospital of Anhui Medical University were analysed. Clinical pathological characteristics (tumour invasion, node, metastasis, and stage) were based on the tumour-node-metastasis (TNM) classification (8th edition), by the International Union Against Cancer.

### Patient selection

The inclusion criteria are as follows: LTESCC without distant metastasis before surgery and no previous neoadjuvant chemotherapy and/or radiotherapy, curative oesophagectomy with lymphadenectomy, pathologically confirmed ESCC, pathological T3-4 with any N stage or N1-3 with any T stage (stage IIa–IVa). The exclusion criteria are as follows: unknown or unclear pathological records, EAC or another type of EC, double or multiple primary cancers, uncertain recurrence, unknown lymph node status, unknown clinical target and dose for PORT, follow-up time less than 2 years.

### Diagnosis of recurrence

The diagnosis of LNM was mainly based on CT, and occasionally MRI or PET/CT was used. Fine needle aspiration was carried out for some instances of cervical LNM. Measurements of the short lymph node diameter > 10 mm in CT/MRI images (5 mm for lymph nodes of the tracheoesophageal groove), fusion of lymph nodes, or the size of the lymph node combined with hoarseness or cough was considered LNM, while the HUVmax value of lymph nodes > 2.4 in PET/CT images was considered LNM. Anastomotic recurrence should be verified by oesophagoscopy. The diagnosis of haematological recurrence was based on imaging diagnosis of different sites.

### Follow-up

Patients were followed up every 2–4 months after surgery in the first 2 years and every 3–6 months thereafter. Re-examinations included chest-enhanced CT scans, abdominal and cervical ultrasound screening, or enhanced CT. When necessary, PET/CT, endoscopy, and fine needle aspiration was performed.

### Statistical analysis

Statistical analysis was performed using the statistical package SPSS (version 19.0 for Windows, IBM SPSS, Armonk, NY, USA). Chi-square tests and Fisher’s exact tests were used for categorical variables. Student’s *t* test was used for continuous variables. Survival was performed using the Kaplan-Meier method, and the results were compared by the logrank test. Univariable and multivariable Cox regression analyses were performed to analyse the risk factors of regional lymph node recurrence. Values of *p* < 0.05 were considered to indicate a statistically significant difference.

## Results

### Patients and treatment regimen

A total of 127 patients with LTESCC were recruited. The patients were divided into four groups: surgery alone (S), surgery with adjuvant chemotherapy (S + CT), surgery with adjuvant radiotherapy (S + RT), and surgery with adjuvant chemoradiotherapy (S + CRT). The average age was 61.02 ± 8.10 years, and the average length of the tumour was 4.34 ± 1.48 cm. The general characteristics of patients at the time of surgery and the characteristics of the adjuvant therapies are summarised in Table 1. There are no distribution differences of the characteristics for S vs. S + CT, S vs. S + RT, and S vs. S + CRT, except age for S vs. S + CRT (*p* = 0.03).

**Table 1:** Characteristics of patients

Parameter	Number (*n* = 127)	Adjuvant therapies						
		S (*n* = 32)	S + CT		S + RT		S + CRT	
			(*n* = 23)	*p* value	(*n* = 26)	*p* value	(*n* = 46)	*p* value
Sex				1.00		0.95		0.78
Male	111	27	20		23		41	
Female	16	5	3		3		5	
Age (years)				0.12		0.38		0.03
< 60	59	10	12		11		26	
≥ 60	68	22	11		15		20	
Length (cm)				0.77		0.86		0.19
< 4	64	14	11		12		27	
≥ 4	63	18	12		14		19	
T stage				0.14		0.43		0.82
T1	9	4	1		1		3	
T2	22	4	8		4		6	
T3	94	23	14		21		36	
T4	2	1	0		0		1	
Nodal stage				0.63		0.10		0.80
N0	41	10	4		14		13	
N1	68	17	16		8		27	
N2	14	3	2		4		5	
N3	4	2	1		0		1	
TNM stage				0.46		0.15		0.82
IIA	41	10	4		14		13	
IIB	11	4	2		1		4	
IIIA	18	3	6		4		5	
IIIB	53	13	10		7		23	
IVA	4	2	1		0		1	
Differentiation				0.94		0.81		0.34
Poor	49	10	7		10		22	
Moderate	71	20	14		15		22	
Well	7	2	2		1		2	

*p* values were used to compare the characteristics of patients for S vs. S + CT, S vs. S + RT, and S vs. S + CRT

Resection via the left chest or right chest and abdomen (Ivor-Lewis) approach with 2FL was used for all patients. The number of lymph nodes dissected was not standardised. All accessible lymph nodes in the mediastinum and upper abdominal area were removed, mainly including the upper and lower paratracheal lymph node, retrotracheal lymph node, subcarinal lymph node, middle and lower paraesophageal lymph nodes, paracardial lymph node, left gastric or celiac lymph node, and the lymph node in the lesser curvature. Anastomosis near the aortic arch was preferred.

The time interval for PORT was 3 to 12 months. The most common (65/73) regimen was 45–56 Gy/25–28 fractions. A dose of 44 Gy/22 fractions or 40 Gy/20 fractions was used for five patients, and a dose of 60 Gy/30 fractions was used for two patients. CT-based planning and a linear accelerator were used to deliver external beam conformal radiation therapy. The clinical target volume (CTV) included the tumour bed with a 3-cm enlargement superiorly and inferiorly, including the upper paratracheal lymph node, lower paratracheal lymph node, subcarinal lymph node, middle and lower paraesophageal lymph node, cardiac lymph node, and the left gastric lymph node. The planning target volume (PTV) was defined as the CTV plus a 0.5–0.8-cm margin.

Combinations of platinum and fluorouracil or/and paclitaxel every 3–4 weeks were used as sequential chemoradiotherapy regimens, while single-agent therapies were not used. Platinum single-agent treatment or combinations of platinum and fluorouracil/paclitaxel every 3–4 weeks were used for concurrent chemoradiotherapy regimens. In our study, more than one cycle of chemotherapy was needed for the chemotherapy groups (S + CT and S + CRT).

### Pattern of recurrence

Eighty-eight patients (69.3%) were identified as having recurrence after oesophagectomy during the minimum 2-year follow-up period. Table 2 shows the recurrence patterns for all patients. The regional lymph node recurrence rates were 68.8%, 82.6%, 50.0%, and 41.3% for the S, S + CT, S + RT, and S + CRT groups, respectively. The anastomosis recurrence rates were 6.3%, 8.7%, 3.8%, and 4.3%, respectively. The haematologic recurrence rates were 25.0%, 21.7%, 26.9%, and 26.1%, respectively. Compared to S, S + RT and S + CRT decreased the recurrence rate significantly (*p* < 0.05), and S + CRT decreased the regional lymph node recurrence rate significantly (*p* < 0.05).

**Table 2:** Pattern of recurrence for different adjuvant therapies

Distribution	S (*n* = 32)	S + CT (*n* = 23)	S + RT (*n* = 26)	S + CRT (*n* = 46)	Total (*n* = 127)
Recurrence	27 (84.4%)	20 (87.0%)	15 (57.7%)*	26 (56.5%)*	88 (69.3%)
Lymph node	22 (68.8%)	19 (82.6%)	13 (50.0%)	19 (41.3%)*	73 (57.5%)
Anastomosis	2 (6.3%)	2 (8.7%)	1 (3.8%)	2 (4.3%)	7 (5.5%)
Haematology	8 (25.0%)	5 (21.7%)	7 (26.9%)	12 (26.1%)	32 (25.2%)
Mixed	5 (15.6%)	5 (21.7%) #	5 (19.2%) #	7 (15.2%)	22 (17.3%)
No recurrence	5 (15.6%)	3 (13.0%)	11 (42.3%)*	20 (43.5%)*	39 (30.7%)

#Recurrence was found in the lymph nodes, anastomosis, and haematologically at the same time. *The recurrence rate had a statistically significant difference compared to S (*p* < 0.05)

### Distribution of regional lymph node recurrence

**Fig. 1:**
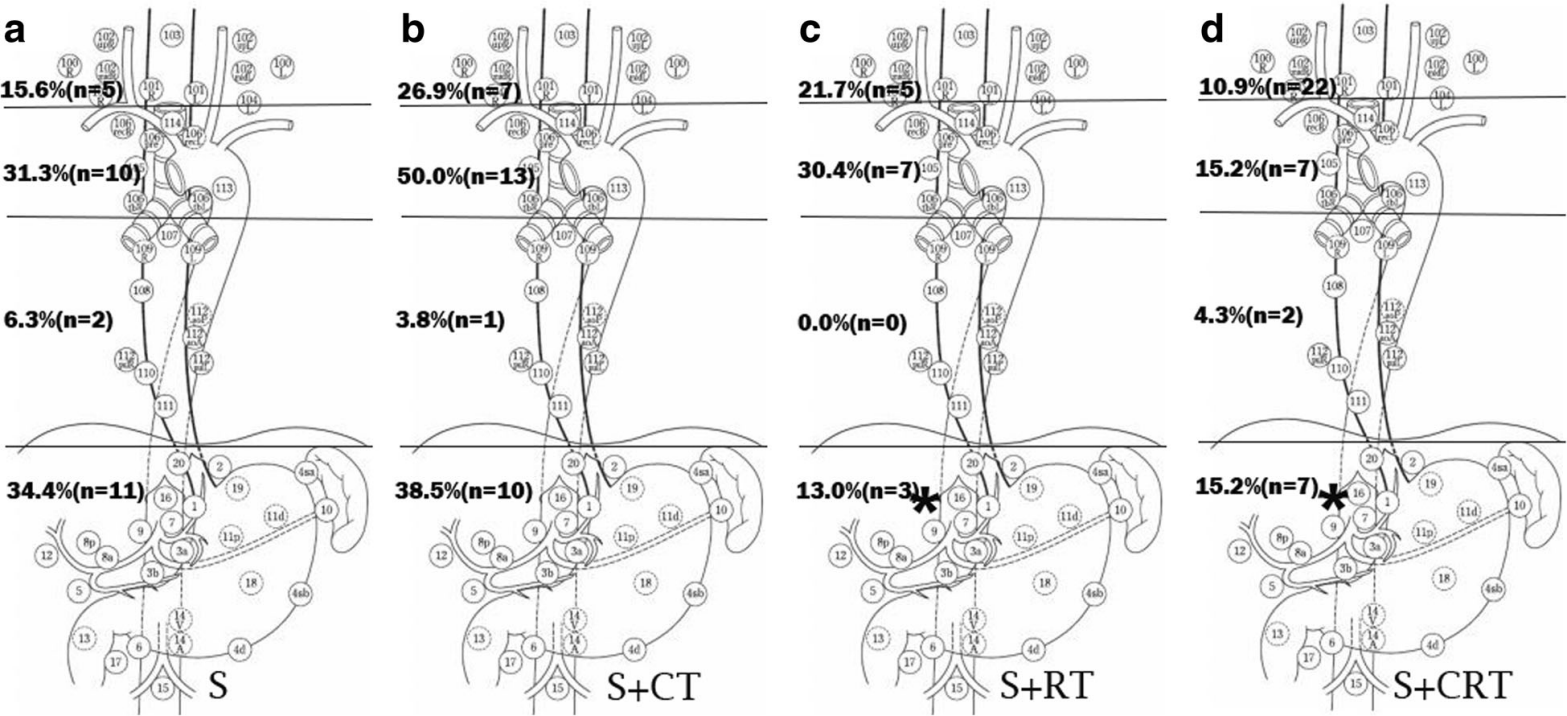
The recurrence rate of different regional lymph nodes after surgery for S(**a**), S+CT(**b**), S+RT(**c**) and S+CRT(**d**) during a minimum 2-year follow-up. *The recurrence rate had a statistically significant difference compared to S (*p*<0.05)

The nodal stations were based on the Japanese Classification of Oesophageal Cancer [25]. The distribution of regional lymph node recurrence is shown in Fig. 1. The lymph node recurrence rates in the neck were 15.6%, 26.9%, 21.7%, and 10.9%, respectively, for S, S + CT, S + RT, and S + CRT. The lymph node recurrence rates were 31.3%, 50.0%, 30.4%, and 15.2% in the upper mediastinum, 6.3%, 3.8%, 0.0%, and 4.3% in the lower mediastinum, and 34.4%, 38.5%, 13.0%, and 15.2% in the upper abdomen for S, S + CT, S + RT, and S + CRT, respectively. Compared to S, only the recurrence rate in the abdomen decreased significantly with S + RT and S + CRT (*p* < 0.05).

### Survival

**Fig. 2:**
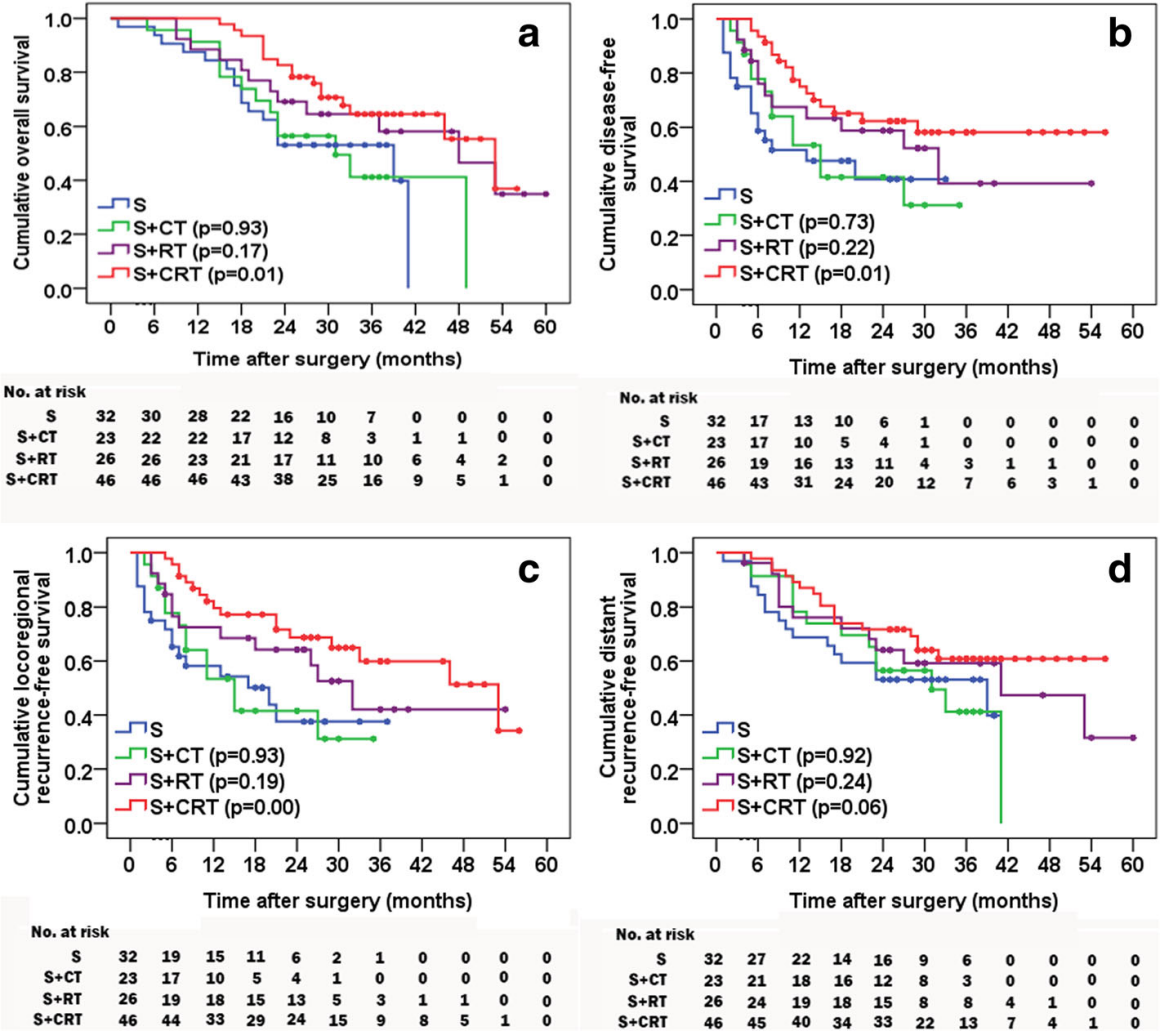
OS (**a**), DFS (**b**), LRRFS (**c**), and DRFS (**d**) for the four groups. *p* values were used for statistical analysis compared to S

The follow-up time was 24 to 60 months (median 39 months) for all patients. For S, S + CT, S + RT, and S + CRT, the median OS times were 39, 31, 48, and 53 months, the 1-year survival rates were 87.5%, 91.3%, 88.5%, and 100.0%, and the 3-year survival rates were 53.1%, 41.2%, 64.6%, and 64.6%, respectively. The OS was statistically different between the S + CRT and S groups, whereas the difference had no statistical significance for S + CT and S + RT compared to S (*p* = 0.93, 0.17, respectively) (Fig. 2a). The disease-free survival (DFS) and locoregional recurrence-free survival (LRRFS) (Fig. 2b, c) for S + CRT did significantly differ compared to S (*p* = 0.01 and 0.00), whereas there were no significant differences for S + CT or S + RT compared to S. There were no significant differences in distant recurrence-free survival (DRFS) (Fig. 2d) for S + CT, S + RT, or S + CRT compared to S (*p* = 0.92, 0.24 and 0.06, respectively).

### Risk factors related to regional lymph node recurrence

As shown in Table 3, the univariable analysis showed that only pathologically positive lymph nodes (PPLNs) located in the lower mediastinum presented a potential risk factor for cervical lymph node recurrence (*p* < 0.05, HR 2.97, 95%CI 1.19–7.39). Univariable and multivariable analyses showed that radiotherapy (HR 0.32, 95%CI 0.15–0.71 and HR 0.30, 95%CI: 0.13–0.68) and PPLNs in upper mediastinum (HR 3.33, 95%CI 1.23–9.06 and HR 3.72, 95%CI 1.30–10.67) were independent risk factors for upper mediastinal lymph node recurrence (*p* < 0.05). Univariable analysis showed that TNM stage (HR 2.87, 95%CI 1.76–7.01), radiotherapy (HR 0.36, 95%CI 0.16–0.83) and PPLNs in the abdomen (HR 2.59, 95%CI 1.15–5.85) were potential risk factors for abdominal lymph node recurrence (*p* < 0.05). However, the multivariable analysis showed that only radiotherapy (HR 0.37, 95%CI 0.16–0.85) and PPLNs in the abdomen (HR 2.57, 95%CI 1.12–5.92) were independent risk factors.

**Table 3:** Univariable and multivariable Cox regression analyses for regional lymph node recurrence

Characteristic	Neck		Upper mediastinum				Abdomen			
	Univariable		Univariable		Multivariable		Univariable		Multivariable	
	HR	95%CI	HR	95%CI	exp(B)	95%CI	HR	95%CI	exp(B)	95%CI
Age (≥ 60 v < 60)	0.64	0.26–1.55	1.02	0.47–2.23	–	–	0.47	0.21–1.06	–	–
Sex (male v female)	2.48	0.75–7.98	0.56	0.15–2.16	–	–	2.10	0.69–6.43	–	–
T stage (T1–2 v T3–4)	0.45	0.18–1.17	0.68	0.29–1.60	–	–	2.92	0.94–9.11	–	–
N stage (N0 v N1–3)	2.80	0.89–8.82	1.42	0.61–3.31	–	–	2.11	0.83–5.36	–	–
TNM stage (II v III + IV)	2.52	0.92–6.86	1.01	0.49–2.33	–	–	*2.87*	*1.76–7.01*	–	–
Length (≥ 4 cm v < 4 cm)	1.10	0.44–2.75	0.93	0.43–2.04	–	–	0.84	0.38–1.89	–	–
Tumour emboli (yes v no)	0.76	0.16–3.68	1.60	0.49–5.26	–	–	1.30	0.37–4.55	–	–
CT (yes v no)	1.27	0.52–3.12	1.20	0.56–2.60	–	–	1.25	0.56–2.77	–	–
RT (yes v no)	0.69	0.28–1.68	*0.32*	*0.15–0.71*	*0.30*	*0.13–0.68*	*0.36*	*0.16–0.83*	*0.37*	*0.16–0.85*
Positive LN in										
UM (yes v no)	1.17	0.35–3.92	*3.33*	*1.23–9.06*	*3.72*	*1.30–10.67*	0.73	0.22–2.37	–	–
LM (yes v no)	*2.97*	*1.19–7.39*	1.24	0.56–2.71	–	–	0.67	0.29–1.57	–	–
Abdomen (yes v no)	0.92	0.37–2.66	1.00	0.46–2.16	–	–	*2.59*	*1.15–5.85*	*2.57*	*1.12–5.92*

*CT* chemotherapy, *RT* radiotherapy, *LN* lymph node, *UM* upper mediastinum, *LM* lower mediastinum

There were statistically significant differences for data in Italics (*p* < 0.05)

## Discussion

We retrospectively analysed data from 127 patients with resected stage IIa-IVa LTESCC with a minimum 2-year follow-up. Locoregional recurrence was the main result of treatment failure after radical surgery with 2FL followed by distant recurrence (approximately 25%), which was similar to other results for thoracic ESCC [16–18]. Postoperative radiotherapy or chemoradiotherapy should be an effective method to prevent decrease recurrence. Chemotherapy not only can control systematic cancer metastasis but can also exhibit a radio-sensitising effect when concurrent chemoradiotherapy is used. Many studies have concluded that aCRT can improve survival for advanced ESCC [12, 13, 15]. There were only a few studies that evaluated the efficiency of adjuvant chemotherapy, and most of these studies showed that aCRT might improve survival only for patients with PPLNs [26]. In our study, adjuvant chemotherapy alone showed no beneficial effects on OS, DFS, LRRFS, or DRFS. PORT provided good survival for stage III and node-positive EC [27, 28]. A recent study showed that PORT using conformal radiotherapy was strongly associated with improved OS and DFS for pT3N0M0 ESCC [14]. In our study, although only the outcomes of OS, DFS, or LRRFS for S + CRT had statistically significant differences compared to S, patients who received S + RT or S + CRT had better OS, DFS, LRRFS, and DRFS than those who received S.

The main type of locoregional recurrence was lymph node recurrence (Table 2). The upper abdomen, upper mediastinum, and lower neck had high recurrence rates (Fig. 1), which are similar to other results [20, 21, 23]. The main lymph flow for the lower oesophagus is descending to the upper abdomen [29]. However, ascending lymph flow to the upper mediastinum and neck is also common due to the intramural bidirectional drainage and long drainage territory [30, 31], which is different from that of the gastric cardia [32]. Furthermore, the descending lymphatics should be terminated at or near the venous angle by the thoracic duct. As a result, lymphatic drainage to Virchow’s nodes could be another route of lymph flow to the cervical nodes. To our knowledge, almost all previous postoperative targets included the lower mediastinum and sometimes the perigastric area for LTESCC. In fact, the lymph nodes in the lower mediastinum and primary perigastric area can be easily removed due to the anatomical features. The recurrence rate was low in these areas after radical surgery [23, 24, 33], which is quite different from the LNM rate of LTESCC found by pathological analysis. The recurrence rate in the lower mediastinum was less than 6.5%, and it was 0.0% in the primary perigastric area in our study. The paraaortic lymph nodes, the truncus coeliacus, the posterior surface of the pancreatic head, and the arteria hepatica communis lymph nodes were the main sites of recurrence in the abdomen after surgery for EC [24]. In our study, the site of abdominal recurrence was mostly in the retroperitoneal region (90.3%). Therefore, we should pay more attention to these areas for abdominal PORT. In our study, the rate of upper abdominal recurrence significantly decreased with S + RT and S + CRT (*p* < 0.05). However, the retroperitoneal region, which was a high recurrence area, was not the main clinical target. The decrease in infield recurrence rate and peripheral low dose irradiation are possible explanations. More evidence is needed for a suitable abdominal target. Regardless of the use of 2FL or 3FL for oesophagectomy, a complete lymph node dissection seems to be difficult in the neck and upper mediastinum because of the complex anatomy and limited surgical field. In our study, though the recurrence rate in the upper mediastinum seemed to decrease after PORT, there was no statistical difference. A larger sample size is needed to better evaluate the differences among treatments. There was a high cervical lymph node recurrence rate, indicating that this region should be a potential target, which may be a limitation for our study. The anastomosis recurrence rate was 6.3% in our study and 6.5% in another study [23]. Hence, it may be an unessential target for PORT due to some potential treatment complications, such as anastomotic fistula and stenosis. Taken together, these results show that the CTV for PORT of LTESCC should focus on the lower neck, the upper mediastinum, and the abdomen.

EC is characterised by bidirectional and skipping LNM, mainly due to the longitudinally mucosal and submucosal lymphatic drainage [34–37]. Collateral vessels of lymph nodes were found because of the close topographical relationship between the afferent and efferent [38], which should be another reason for the skipping LNM. As a result, the lymphatic drainage of the oesophagus is not from the inner layer to the outer layer and from the nearby nodes to the distant nodes step by step. In our study, the pathological T stage (depth of tumour invasion) was not a risk factor for recurrence in all three regions (Table 3). The regions of PPLNs rather than the pathological N stage were risk factors for specific regional recurrence. Due to the ascending mediastinal lymphatic system, PPLNs in the lower mediastinum may be a risk factor for LNM of the neck and the upper mediastinum for LTESCC. In our study, PPLNs in the lower mediastinum was a potential risk factor for cervical recurrence while it was not a risk factor for the upper mediastinal recurrence. Because 2FL were carried out for all the patients, lymph nodes in the neck were not cleaned up while lymph nodes in the upper mediastinum were mostly cleaned up. This should be a major reason. The celiac LNM was mainly through the submucosal direct drainage and partly the descending extramural lymphatic drainage near the abdominal oesophagus. Therefore, PPLNs in the mediastinum (upper or lower) was not a risk factor for celiac recurrence while PPLNs in abdominal region was an independent risk factor for abdominal recurrence. In a similar way, PPLNs in the upper mediastinal regions should be a risk factor for the cervical LNM. However, PPLNs in the upper mediastinum was only an independent risk factor for the upper mediastinal recurrence while it was not a risk factor for the cervical recurrence in our study. More patients should be enrolled to verify these results. Radiotherapy was an independent risk factor for the upper mediastinal recurrence and abdominal recurrence in our results, suggesting the effect of radiotherapy for controlling locoregional recurrence. However, we cannot evaluate the effect of radiotherapy for the cervical region due to its exclusion in the target. These results may provide more evidences for the individual target of PORT.

## Conclusion

S + CRT demonstrated a significantly better OS, DFS, and LRRFS for resected stage IIa–IVa LTESCC. Lymph node recurrence was the main cause of treatment failure after radical oesophagectomy, and the recurrence nodes were mostly distributed in the neck, upper mediastinum, and upper abdomen. These regions may be high-risk targets for PORT. PPLN regions may be important factors for individual targets. Prospective controlled studies with more suitable adjuvant therapies are needed to confirm these results in the future.

## Abbreviations

2FL: Two-field lymphadenectomy
3FL: Three-field lymphadenectomy
aCRT: Adjuvant chemoradiotherapy
CTV: Clinical target volume
DFS: Disease-free survival
DRFS: Distant recurrence-free survival
EAC: Oesophageal adenocarcinoma
EC: Oesophageal carcinoma
ESCC: Oesophageal squamous cell carcinoma
LNM: Lymph node metastasis
LRRFS: Locoregional recurrence-free survival
LTESCC: Lower thoracic oesophageal squamous cell carcinoma
nCRT: Neoadjuvant chemoradiotherapy
OS: Overall survival
PORT: Postoperative radiotherapy
PPLN: Pathologically positive lymph node
S + CRT: Surgery with adjuvant chemoradiotherapy
S + CT: Surgery with adjuvant chemotherapy
S + RT: Surgery with adjuvant radiotherapy
S: Surgery alone
